# Constructing a profound experience model of Chinese Intangible Cultural Heritage Tourism based on grounded theory

**DOI:** 10.1371/journal.pone.0351084

**Published:** 2026-06-11

**Authors:** Ming Lou, Shaomei Hu, Di Hu, Qing Zhang

**Affiliations:** Academy of Art and Design, Anhui University of Technology, Ma’anshan, China; Guilin University of Technology, CHINA

## Abstract

The purpose of this paper is to understand the generative mechanism of the profound experience in Chinese Intangible Cultural Heritage (ICH) tourism and its construction of meaning, to enrich the research on cultural tourism experiences, and to provide theoretical support for the sustainable development of Chinese ICH tourism. The study employs a Grounded Theory approach to summarize the theoretical model of profound experiences in Chinese ICH tourism, based on the network text descriptions of typical profound experiences in Chinese ICH tourism. The results of the study indicate that:(1) The theoretical model of profound experience in Chinese ICH tourism consists of four main categories: basic conditions, perceptual evaluation, personal significance, and social significance, along with 15 corresponding subcategories. (2) Professional service touchpoints, experience content innovation, scene atmosphere creation, application of modern technology, participation in interactive socialization are the basic conditions for the generation of profound experiences in ICH tourism. The dimensions of perceptual evaluation, such as sense of participation, sense of wonder, sense of immersion, sense of resonance, and sense of value, can describe the profound experiences obtained by tourists. Cultural cognition, cultural identity, and cultural memory reflect the personal significance achieved through the profound experience of ICH tourism, while the inheritance and dissemination of ICH reflect its social significance. The findings of this paper highlight the importance of profound experiences in enhancing the quality of cultural tourism and offer insights to Chinese tourist attractions, assisting them in improving tourism design in aspects such as tourism products, service systems, and scenarios, thereby elevating the overall experience quality of ICH tourism.

## 1. Introduction

Intangible Cultural Heritage Tourism (ICHT), a form of cultural tourism centered on the utilization of intangible cultural heritage resources [1], not only enriches the cultural offerings of tourism but also allows tourists to engage with the spiritual essence of ancient life-styles and cultural heritage. This is achieved through their cognitive, participatory, and educational involvement with intangible cultural heritage during their visits [2]. For instance, the Nanjing Qinhuai Lantern Festival Folk Customs Tour and the Jingdezhen Ceramic Firing Technique Experience Tour have attracted significant numbers of visitors, establishing themselves as prominent ICH tourism destinations in China.

Tourism experiences have become a significant psychological need throughout the tourism process. Scholars such as Xie Yanjun and Su have argued that tourism experiences represent the core of tourism [3,4]. Xu Hong and Li Qiuyun have categorized tourism experience quality into hierarchical dimensions, including physiological, emotional, and cognitive experiences [5]. The quality of tourism experiences has emerged as a key indicator for enhancing tourist satisfaction and ensuring sustainable participation in tourism activities [6]. When embodied and integrated experiences surpass single-sensory experiences, the depth, intensity, and scope of tourism experiences are significantly enhanced, prompting tourists to seek higher-level experiences. McKercher identifies profound experiences as an essential component of cultural tourism [7]. The distinctive characteristics of ICH tourism—its dynamic nature, cultural richness, and educational value—ensure that visitors’ deep perception of and resonance with ICH culture form the core of its profound experiences. By deeply engaging in cultural activities, actively participating in cultural interactions, and acquiring a profound understanding of cultural significance, travel experiences transcend superficial dimensions related to aesthetics and functionality, reaching deeper dimensions associated with emotion, meaning, and value. This process can enhance tourists’ cultural participation and cultural identity during travel, increase their recognition of the value of Intangible Cultural Heritage (ICH), and ultimately improve the overall quality of cultural tourism experiences. Although some scholars have examined the connotations of travel experiences and the mechanisms influencing tourism experience quality, specialized studies on the profound experiences within Chinese ICH tourism remain relatively scarce.

Currently, the development of ICH tourism continues to face challenges such as superficial experiences, limited visitor engagement, and pronounced commercialization. Many ICH tourism projects remain confined to static displays and performance-oriented sensory experiences, leaving visitors without a profound understanding or emotional resonance with the heritage culture. This not only diminishes visitors’ perception of the heritage itself but also reduces satisfaction and weakens the destination’s overall appeal. Amid the deep integration of culture and tourism, visitors increasingly seek profound insights and resonance with ICH—a central focus of research on profound-level ICH tourism experiences. Taking Jingdezhen porcelain craftsmanship tourism as an example, earlier offerings were largely limited to sightseeing and observation, resulting in superficial visitor experiences. Today, however, visitors can participate in ceramic workshops, immerse themselves in the crafting process, and engage in meaningful interactions with ICH bearers, thereby achieving more profound experiential gains during their travels. This enhances visitors’ understanding and appreciation of ICH, elevates the quality of their tourism experiences, and fosters a stronger willingness to share and revisit. Such practices play a vital role in preserving ICH and promoting the sustainable development of tourism.

From a theoretical perspective, frameworks such as Experience Economy Theory, Transformative Tourism, and Cultural Identity Theory all emphasize the centrality of experience. Through cultural participation and interaction, these theories suggest that individuals can undergo positive transformations in lifestyle, cultural identity, and experiential value. This aligns closely with the pursuit of profound experiences in ICH tourism. The insights derived from these theoretical perspectives provide a foundation for examining the essence of profound ICH tourism experiences and the mechanisms influencing tourism experience quality in this study. Recent academic research has explored various aspects of cultural and ICH tourism experiences. For instance, Seyfi et al. developed the Memorable Cultural Tourism Experience (MCTE) theoretical model [8]; Wang and Li investigated virtual experience models and digital preservation pathways for ICH tourism using metaverse technology as an entry point [9]; Yu emphasized the emotional and ritualistic dimensions of ICH tourism experiences [10]; and Cuomo et al. examined how social big data can be applied to cultural tourism experiences through co-design to generate additional value for tourists [11]. Despite the growing body of re-search on cultural and ICH tourism experiences, most studies remain focused on techno-logical empowerment, local identity, and superficial perceptions. There is still limited exploration of profound experiences—particularly tourists’ cultural resonance, emotional immersion, and meaning construction—especially within the Chinese context. Research specifically addressing profound experiences in ICH tourism lacks systematic theoretical frameworks and empirical evidence. Therefore, further investigation is needed into the conceptualization, generative mechanisms, and meaning construction of profound experiences in China’s ICH tourism.

The Grounded Theory approach focuses on deriving universal principles from empirical data. Through systematic analysis and coding of raw materials such as interview transcripts and online reviews, it inductively develops concepts and theoretical frameworks, making it particularly suitable for research fields where mature theories have yet to be established [12]. A distinctive feature of the tourism industry is that travelers frequently share their experiences and impressions on public platforms, providing abundant data sources. Researchers can apply Grounded Theory to synthesize these publicly available data. Numerous studies have adopted this approach to examine attitudes toward tourism activities [13], memorable travel experiences [14], and perceived evaluations of tourist attractions [15]. Given the scarcity of research on profound experiences in ICH tourism and the absence of established models, Grounded Theory offers a systematic means of inducing concepts and frameworks from qualitative data (e.g., user-generated content and interview transcripts). Accordingly, this study employs Grounded Theory as its primary methodology to construct a systematic research framework for profound experiences in ICH tourism.

Therefore, to address the aforementioned research questions, this study proposes the following research objectives: (1) to clarify the connotation and occurrence mechanism of profound experiences in Intangible Cultural Heritage (ICH) tourism; (2) to summarize the perceptual evaluation characteristics of profound experiences in ICH tourism; and (3) to explore the processes and mechanisms underlying the construction of meaning in profound experiences within ICH tourism. To achieve these objectives, this study analyzes user-generated content related to ICH tourism experiences from Chinese social media platforms and adopts Grounded Theory methodology to investigate the mechanisms underlying profound experiences in Chinese ICH tourism. Additionally, it examines how tourists attain cultural cognition, identification, and memory through profound experiences, and how ICH culture is preserved and disseminated through such tourism practices. Theoretically, this study defines the concept of profound experiences in Intangible Cultural Heritage (ICH) tourism, reveals the mechanisms underlying the formation of profound experiences in Chinese ICH tourism, and elucidates the logic of meaning construction, thereby contributing to the enrichment of the theoretical framework of cultural tourism experiences. Practically, this study offers guidance for the sustainable development of ICH tourism and holds significant practical value for enhancing the quality of ICH tourism experiences.

## 2. Literature review

### 2.1 Intangible Cultural Heritage Tourism

Intangible Cultural Heritage (ICH) tourism is a representative form of cultural tourism, in which ICH is utilized as a tourism resource and tourism activities are conducted with the aim of satisfying tourists’ specific cultural needs [16]. Current research in this field primarily concentrates on topics such as resource development, tourism planning, cultural transmission, and cultural perception. Among these, ICH tourism—with its distinctive characteristics of cultural exploration and cultural experience—has diversified the public’s means of perceiving ICH [17]. Liu Tuo identified the display formats of process-based and dynamic presentations in ICH tourism [18], while Ma Xiaona et al. categorized two immersive perception approaches: museum-based displays and art design-based displays [19]. Li Xijian suggested leveraging cultural creativity to activate the aesthetic value of ICH resources in tourism [20], thereby fully enhancing their cultural appeal. Driven by advancements in new technologies, gamified interaction, integrated media communication, and digital space exploration have introduced novel approaches for perceiving ICH [21,22]. In the era of the experience economy, ICH tourism is developing along the trajectory of primary products → products → services → experiences [23], which not only reflects the diversity of regional cultures but also provides tourists with meaningful travel experiences [24]. This process forms a positive influence pathway of “travel experience – travel emotions – cultural identity” in shaping emotional responses during tourism experiences [25].

### 2.2 Travel experience

Experience represents the essence of tourism, while culture constitutes its core content and defining characteristic. In 1979, Cohen examined tourism experiences from a phenom-enological perspective, highlighting the diverse meanings these experiences hold for travelers [26], thereby initiating scholarly interest in the study of tourism experiences. Since the 1990s, researchers have incorporated theoretical frameworks such as Embodied Experience theory, Ritual Theory, Symbolic Interaction theory, and Phenomenology to explore the fundamental nature of tourism experiences [27,28]. In the 21st century, the centrality of “experience” in tourism research has become increasingly evident. Scholars such as Xie Yanjun, MacCannell, and Ryan have conducted more indepth investigations into the connotations, motivations, and processes of tourism experiences [29–31]. Although consensus has not yet been reached regarding the core factors influencing tourism experiences, it is generally agreed that such experiences reflect the value of tourism products and contribute to tourists’ internal satisfaction, pleasure, and enjoyment [32,33].

Tourists, due to differences in personal background, interests, and travel motivations, experience variations in the quality of their travel experiences. In cultural tourism research, service touchpoints, tourism product innovation, and onsite interactions are regarded as critical nodes for interpreting cultural essence and enhancing experiential quality [34,35]. These elements encompass multiple stages, including tour reservations, cultural guided tours, and dining services [36], collectively shaping visitors’ multidimensional perceptions of cultural content. Such an approach deepens visitors’ cognitive engagement and emotional investment [37]. Most researchers agree that extracting key characteristics from tourism services to assess experience quality offers a better under-standing of tourists [5]. By identifying the factors influencing travel experience quality and defining the evaluation dimensions of experience quality, it is possible to establish relationships between experience quality and tourist satisfaction, perceived image, perceived value, and behavioral intentions [38–40]. Gao et al., based on consciousness spectrum theory, classified tourism experiences into five types: sensory experiences, cognitive experiences, emotional experiences, return experiences, and spiritual experiences [41]. Filep et al. summarized the positive effects of tourism experiences, including meaningful activities, positive interpersonal relationships, a sense of life meaning, and personal achievement fulfillment [42]. Researchers frequently apply Grounded Theory methods to examine tourism experiences, such as tourism perceptions and expectations [43], factors influencing tourism experience quality [44], and sustainable tourism development in heritage sites [45]. This methodological approach generates theory from empirical data, providing an irreplaceable advantage in exploring complex and dynamic social phenomena where mature theoretical frameworks are lacking. It is particularly well-suited for studies on tourism experiences and heritage, serving as an effective methodological tool for investigating profound experiences in ICH tourism. In this study, Grounded Theory can be applied since there is no unified standard for the classification of tourism experience quality dimensions in the academic community. Particularly in the context of cultural tourism experience research, it is necessary to establish more targeted dimensions to differentiate various experience out-comes and further investigate the relationships among variables, such as the conditions for generating profound experiences, perceptual outcomes, and meaning construction.

### 2.3 Profound experiences in Intangible Cultural Heritage Tourism

Profound experiences are considered an extension of experience design theory. In 2014, Jensen distinguished the dimensions of profound experiences—tool dimension, usage dimension, and deep dimension—in his article “Designing for profound experiences” [46], which is regarded as the first formal definition of the concept of “profound experiences” in the field of design studies. Jensen identifies meaning as a critical interactive quality indicator in profound experiences, addressing users’ higher-order experiential needs related to value and spirituality, thus yielding a stronger sense of experiential fulfillment with social attributes [47–50]. Hassenzhal and Desmet argue that experience design should focus on providing moments of meaning [51,52], which helps foster the interconnectedness of users’ physical, psychological, and environmental aspects, ultimately achieving a more profound experiential sensation [53]. Scholars have applied profound experience theory in fields such as public services, community innovation, and game design to explore museum service experience design [54], persuasive game design [55], and workplace well-being experience design [56]. While some scholars have noted the profound emotional responses in travel experiences [57], the dimensions of profound experiences vary significantly across different scenarios and types of travel, necessitating further analysis of their underlying mechanisms.

Compared to general tourism, Intangible Cultural Heritage (ICH) tourism exhibits a more pronounced social attribute in terms of cultural dissemination and education. Tourists often need to engage in hands-on participation, immersive appreciation, and in-depth interaction to fully understand the essence of ICH culture. For example, the Lijiang Dongba Papermaking Study Tour emphasizes cultural empathy, meaning creation, and value realization, which are precisely the key components of profound experience research in ICH tourism. In previous studies by Jensen and the author, sensory-level and behavioral-level experiential feelings were defined as shallow experiences. Jensen categorized experiential feelings related to the meaning dimension as profound experiences, while the author defined experiential feelings associated with the emotional, social, and value dimensions as profound experiences, aiming to create multi-layered immersive experiences, positive life impacts, personal value realization, and social value integration for users [58].

Based on previous research by Jensen and the author, the profound experiences in ICH tourism referred to in this study pertain to the experiences that tourists acquire when they fully immerse themselves in the cultural context of ICH during participation in such tourism activities. This involves gaining a profound understanding of the cultural connotations, historical origins, and technical essence of ICH, and through a deep perception of ICH, obtaining experiences related to personal emotions, cultural significance, and cultural value. Shallow experiences in ICH tourism may provide temporary pleasure through behavioral satisfaction or cognitive fulfillment, while profound experiences can have significant impacts on tourists’ emotional connections, cultural identity, meaning attainment, and value transformation. Ad-ditionally, shallow experiences often serve as the foundation for profound experiences, with the latter representing the elevation of the former. Tourists can gradually transition from shallow to profound experiences through scene creation, cognitive deepening, and emotional interaction in ICH tourism. It should be noted that this study focuses on promoting the achievement of experiences at the emotional, meaningful, and value levels, as well as the performance of experience achievement within the framework of profound experiences, rather than exploring sensory or behavioral aspects of the experience content.

### 2.4 Conceptual anchors for the profound experience model

Research on the experience economy theory, tourism experience studies, and ICH preservation theory has examined variables and elements within related models such as ser-vice experiences, tourism experiences, and ICH dissemination. Zomerdijk et al. argue that creating unique, enjoyable, and memorable experiences constitutes the core objective of product or service design [59]. As service processes in tourism, commerce, and community sectors become increasingly specialized, Verhoef et al. identify “service touchpoints,” “social environment,” and “scenario atmosphere” as key experiential drivers, emphasizing that staff professionalism and interaction quality significantly influence customer emotions and value assessments [60]. Liu positions employee service capabilities, touchpoint design, and tour route management as critical experiential drivers, highlighting that professional performance and interaction quality of staff substantially affect customer emotions and value evaluations [61]. Schmitt’s experience marketing theory advocates incorporating novel, narrative, and interactive content to enhance customers’ experiential engagement and memory retention toward brands and scenarios [62]. Lucas, Ryu, and others examined the combined influence of environmental ambiance on sensory and emotional experiences, underscoring its vital role in improving experience quality [63,64]. Furthermore, immersive technologies such as VR, AR, and MR have been widely applied in cultural heritage contexts, not only enhancing visitors’ sense of presence and interactivity but also improving their understanding of heritage information and learning outcomes [65]. Some researchers suggest that tourism experiences are inherently social, wherein user participation and interpersonal interaction not only align with the concept of co-creating service experience value [66] but also significantly enhance travel enjoyment [67]. Collectively, these studies demonstrate that service touchpoints, experiential content, contextual ambiance, technological applications, and service interactions jointly influence the overall quality of user experiences.

Numerous existing studies indicate that under different triggering conditions, users exhibit distinct psychological and emotional responses, reflected in varied experiential perceptions. Pine and Gilmore categorize experiential outcomes into two dimensions: participation and immersion [68]. Hlava et al. explain profound experiences through immersion, awe, and resonance [69]. McLellan identifies pleasure, immersion, and sense of value as core pursuits in experience design [70]. Desmet and Hekkert propose a positive experience model encompassing aesthetics, value, and virtue, asserting that experience design ultimately aims to create a good and joyful life [71]. In tourism experience research, Otto et al. developed a tourism experience scale comprising hedonism, interactivity, novelty, participation, safety, and excitement [72]. Kim et al. identified 24 factors influencing memorable tourism experiences, categorizing them into seven key dimensions: hedonism, novelty, local culture, freshness, meaning, participation, and knowledge [73]. Buonincontri et al. examined primary antecedents and consequences of cocreated tourism experiences, focusing on participation, happiness, and value-related quality indicators [74]. Balaskas et al. developed a cultural heritage experience evaluation scale encompassing engagement, resonance, immersion, and flow [75]. Chandralal et al. found that authentic participation, novelty, interactivity, and perceived meaning influence memorable tourism experiences [76]. Existing research has investigated the enabling conditions for achieving experiences and diverse experiential perceptions; however, variables within these experiential models remain fragmented across different themes. Further research on tourism experience models tailored to specific themes is therefore necessary. These findings provide theoretical references for subsequent variable synthesis.

### 2.5 Critical literature reviews

Intangible Cultural Heritage (ICH) tourism fulfills tourists’ needs for cultural exploration and cultural experiences. However, in the era of the experience economy, enhancing tourists’ emotional responses and psychological satisfaction during the tourism process has become an increasingly important research topic. Scholars have recognized the significance of the cultural nature of ICH in positively influencing the tourism experience. Research findings have demonstrated that variations in tourists’ personal backgrounds, interests, and tourism motivations result in differences in the evaluation dimensions of tourism experience quality. Previous research findings also indicate that the Grounded Theory methodology is well-suited for identifying new categories, mechanisms, and meaning-making processes within ICH tourism. The findings related to profound experiences have been applied to research on higher-level experiential needs across various fields. However, some shortcomings still exist that require further investigation: (1) While the experiential objectives of ICH tourism align with the research framework of profound experiences, the concept and connotations of profound experiences in Chinese ICH tourism need further exploration to identify new manifestations. (2) Chinese ICH tourism is a uniquely distinctive form of cultural tourism, which determines that the generative conditions and perceptual outcomes of profound experiences in ICH tourism also possess distinctiveness, necessitating specialized identification. (3) The meaning construction of profound experiences in Chinese ICH tourism is a complex psychological operational mechanism in-volving the interplay of multiple elements such as service perceptions, experiential achievement, and cultural influence. This requires more targeted research methods to explore and reveal the logical framework and process mechanisms underlying the meaning construction of profound experiences in ICH tourism.

Therefore, this study uses online texts related to profound experiences in ICH tourism in China as the unit of analysis for Grounded Theory research. It aims to explore the conditions that promote the generation of profound experiences in ICH tourism, the perceptual outcomes of achieving these experiences, and how they subsequently influence the quality of cultural tourism experiences. The goal is to deepen the theoretical understanding of ICH tourism experiences in China.

## 3. Research design

### 3.1 Research methods and process

Grounded Theory was first proposed by Glaser and Strauss of Columbia University in their 1967 book The Discovery of Grounded Theory. It is a bottom-up, qualitative research method based on the inductive analysis of empirical data. This approach systematically collects data to identify the core concepts reflecting a particular phenomenon, and uses level coding techniques to extract the categories and classes of relevant phenomena from the original data, ultimately constructing the corresponding theoretical model [77]. Grounded Theory has been widely applied in the fields of user experience and tourism experience [78–81], with many studies forming theories by establishing connections between these core concepts through Grounded Theory.

The generation of profound experiences in Chinese Intangible Cultural Heritage (ICH) tourism is influenced by a multitude of complex factors, making it difficult to summarize a comprehensive and systematic theoretical model based on a single design case. Additionally, this paper adopts the Grounded Theory method for two main reasons: First, research on the “profound experiences of Chinese ICH tourism” is in its early exploratory phase. Grounded Theory provides an effective approach for meticulously organizing raw data, facilitating the corresponding exploratory research. Second, the foundational conditions, perceptual evaluation, and meaning effects involved in the generation of profound experiences in Chinese ICH tourism are characterized by complex, process-oriented relationships. Traditional qualitative and quantitative methods often struggle to address such complexities. However, Grounded Theory can tackle these challenges through a process of level-by-level coding and the construction of narrative storylines. Therefore, this paper employs Grounded Theory to conduct inductive research on the theoretical model of profound experiences in ICH tourism, aligning closely with both the study’s objectives and the characteristics of the re-search subject.

**Fig 1:**
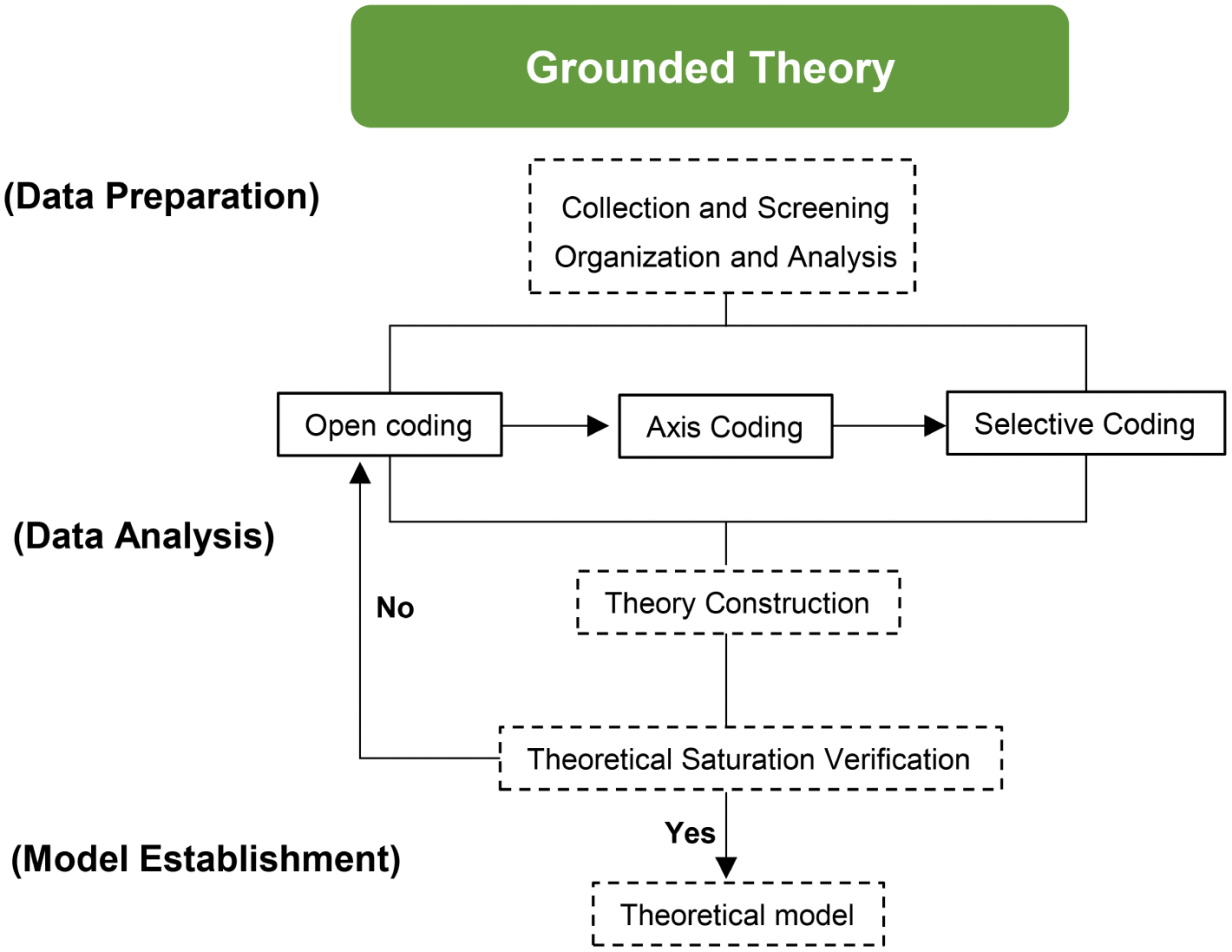
Research framework. Based on the Grounded Theory research method, the research framework mainly consists of three parts: data preparation, data analysis, and model establishment.

**Fig 2:**
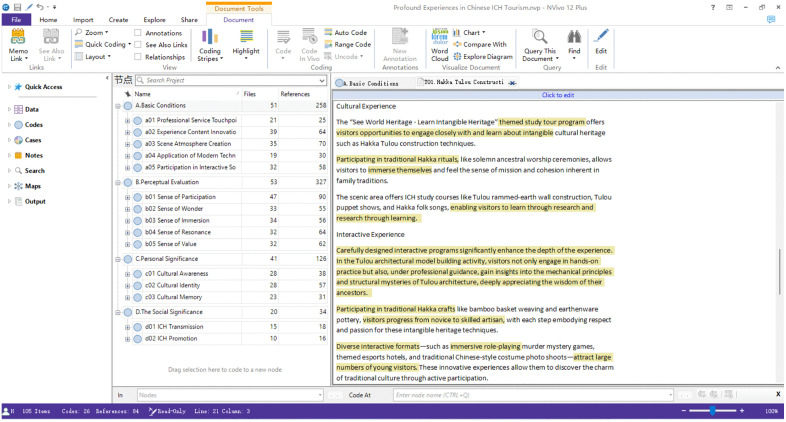
Data coding process using NVivo12 software. The data coding process includes open coding, main axis coding, and selective coding conducted with NVivo12 software.

The research framework, as illustrated in Fig 1, consists primarily of three components: data preparation, data analysis, and model building. The specific steps are as follows:

(1) Data preparation provides a robust foundation for future research and enhances the credibility of the research findings. In the digital age, an increasing number of tourists are sharing their travel experiences on social media, and online texts have gradually become a key data source for scholars exploring travel experiences [82]. This paper utilizes travelogues and reviews related to the profound experiences of Chinese ICH tourism as data sources, offering samples for data analysis.
(2) The data analysis was conducted in three steps: open coding, axial coding, and selective coding. Using the text analysis method, the relevant descriptions of profound experiences were analyzed line by line to identify the core concepts embedded within them. Subsequently, the textual content was coded progressively to abstract, generalize, and refine the concepts, ultimately forming categories. Additionally, a portion of the textual data was reserved for theoretical saturation testing. The coding process was concluded when core concepts repeatedly emerged without generating new concepts. The basic analytical unit in this study is defined as “textual fragments of tourists’ profound experiences with ICH tourism,” refer-ring specifically to relatively complete semantic units within travelogues or review texts that revolve around a single experiential theme. For instance, the sentence “At Shanshui Suzhou Embroidery Gallery, personally experiencing Suzhou embroidery was truly fascinating!” is regarded as a semantic fragment and is coded with initial concepts such as “firsthand experience” and “sense of wonder.” During the coding process, this study strictly adhered to the three-tier coding logic of Grounded Theory. First, in the open coding phase, the researchers analyzed the raw textual data line by line to identify key phrases that reflect tourists’ profound experiences in ICH tourism, thereby generating initial concepts. Second, during the axial coding stage, similar or related concepts were aggregated to form preliminary categories (for example, “contributing one’s efforts,” “willingness to purchase,” and “willingness to participate again” were integrated under the category of cultural identity). Finally, in the selective coding stage, relationships among the existing categories were systematized to extract the core categories of profound experiences in ICH tourism. Logical connections between the core and other categories were established, and a theoretical model was constructed through narrative threads. To enhance research transparency, partial data coding processes (see Fig 2) and coding diagrams (see Table 1) are provided herein.

**Table 1:** Coding reference table (Partial).

Original Text Fragment	Open Coding (Initial Concept)	Main Axis Coding (Category)	Selective Coding (Core Category)
Through hands-on experiences in tea picking, withering, killing green, and rolling, we not only learned traditional tea-making techniques but also felt the historical legacy of Wuyuan tea culture.（T04）	Firsthand Experience	Sense of Participation	Perceptual Evaluation
People can interact with intangible cultural heritage bearers, local artisans, and fellow travelers, sharing their experiences and impressions.（T07）	Interactive Exchanges	Participation in Interactive Socialization	Basic Conditions
She also mentioned that the museum boasts beautiful surroundings and a rich cultural atmosphere, making it a truly unforgettable cultural journey. (T28)	Unforgettable	Cultural Memory	Personal Significance

Prior to formal coding, the researchers conducted small-scale pilot coding on three representative ICH tourism cases (Hakka Tulou Construction Techniques, Xiangxi Miao Drum Dance, and Miao Silver Jewelry Craftsman-ship) to assess the applicability, operability, and consistency of the preliminary coding framework. The pilot results indicated a stable distribution of key concepts, clear coding classifications, and high discriminability among concepts, demonstrating the framework’s robust operability and logical consistency. Based on feedback from the pilot coding, the re-searchers refined the initial coding table, clarified inclusion and exclusion criteria, and particularly elaborated the distinction between the dimensions of Profound and superficial experiences to ensure accuracy and validity in subsequent coding. This pilot coding laid a solid foundation for the smooth implementation of systematic coding.

(3) Model building assists researchers in quickly grasping the key aspects of the study and offers guidance for future research. This step is based on previous analyses and involves exploring the relationships between concepts and categories. A new theoretical model is then constructed in the form of a storyline, summarizing the generative mechanisms, meaningful roles, and future impacts of profound experiences in Chinese ICH tourism.

### 3.2 Data sources and description

**Fig 3:**
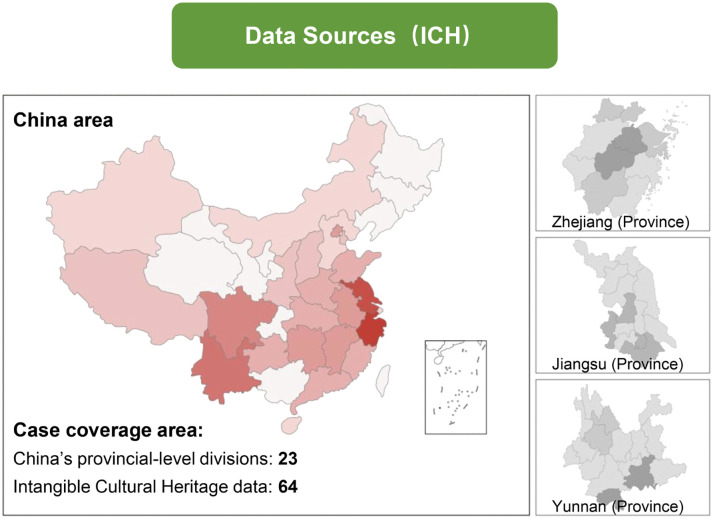
Geographic distribution of study cases. The collection of raw data on ICH tourism cases mainly includes the number of cases and their geographical distribution.

**Fig 4:**
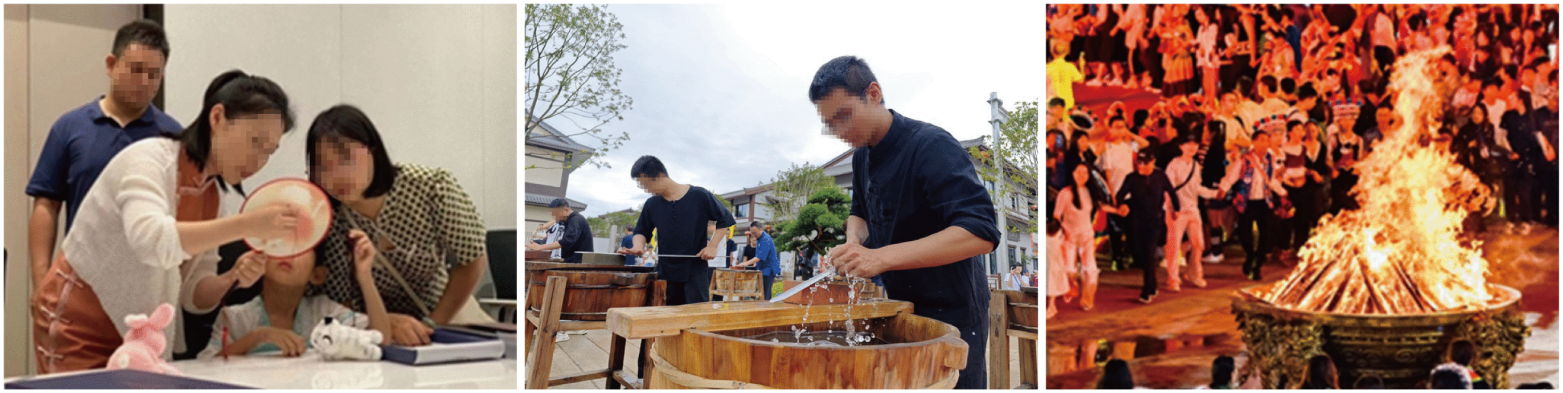
Three representative Chinese ICH tourism programs. A selection of outstanding ICH tourism case studies, including: **(a)** Suzhou embroidery [83] **(b)**Longquan sword-making [84] **(c)**The Yi Torch Festival [85].

This paper collects documents such as travelogues and reviews related to Chinese In-tangible Cultural Heritage (ICH) tourism from several online platforms where Chinese tourists are most inclined to share their travel experiences and feelings. These platforms include travel communities (e.g., Mafengwo, Qunar, Ctrip), blogs (e.g., Zhihu, Douban), and social media (e.g., TikTok, Xiaohongshu). We exclude tourists’ comments related to sensory and behavioral aspects, focusing instead on descriptions and evaluations of tourism experiences at the emotional, social, and value levels. In the process of selecting samples for the study of Chinese Intangible Cultural Heritage (ICH) tourism, the following principles were strictly adhered to: (1) Focus on selecting tourism cases of national-level ICH projects to ensure the authority of the samples;(2) The sample was designed to encompass as many provinces and regions in China as possible to ensure the universality and diversity of the results. A total of 64 ICH tourism cases were selected, covering 23 provinciallevel administrative regions across China (see Fig 3). Regions such as Zhejiang, Jiangsu, and Yunnan accounted for a larger number of cases, primarily due to their abundance of nationally designated ICH projects and their high level of tourism development maturity. (3) Priority was given to selecting well-regarded and popular cases to ensure high-quality samples that are highly relevant to the research theme. Examples include Suzhou embroidery, Longquan sword-making experience, and the Yi Torch Festival experience, all of which are ICH tourism projects that have been particularly favored by tourists in recent years (see Fig 4). (4) During the sample selection process, relevant screening criteria were applied. The inclusion criteria included: (a) projects featuring explicit visitor participation and interactive components; (b) visitor travelogues or review texts containing emotional or cognitive responses to cultural experiences; and (c) projects exhibiting notable levels of online engagement, such as platform reposts and comment volumes. The exclusion criteria included: (a) projects primarily focused on display or spectator activities lacking interactive experiences; and (b) tourism activities centered on commercial performances or festive entertainment with limited cultural depth.

In addition, this study comprehensively considered both theoretical saturation and content diversity in determining the sample size. Based on the findings from preliminary coding, the core concepts stabilized at approximately 50 cases, with no new concepts emerging in the subsequently added cases. This determination established the research sample size, ensuring both theoretical saturation and representativeness. During the data processing stage, we manually reviewed the data to filter out irrelevant descriptions of tourism experiences and ensure the accuracy and relevance of the textual descriptions to the research. After filtering, we retained approximately 76,000 words of relevant descriptions and evaluations from 64 research samples out of the initially collected 90 cases. Of these, 53 samples were coded and analyzed using NVivo12 for data coding, numbered T1–T53, while the remaining 11 items were numbered Y1–Y11 and used for theoretical saturation testing. The verification process was guided by two core criteria: “whether new concepts or categories emerge” and “whether the attributes of existing categories are further enriched.” The results indicate that the coding process did not generate any new significant categories, and the internal characteristics of the existing categories tended toward stability and repetition. This reflects a state of “high coding repetition rate with no emergence of new categories.” Accordingly, it can be concluded that the constructed theoretical model has achieved theoretical saturation and demonstrates good robustness.

This study adopts a qualitative research design to explore the generative mechanisms and meaning construction of profound experiences in China’s ICH tourism. This is accomplished through the analysis of publicly available textual materials—such as user reviews and travelogues—posted on open online platforms including Mafengwo, Zhihu, Xiaohongshu, and TikTok. The study strictly adheres to the ethical standards outlined in the Declaration of Helsinki and received approval from the Ethics Committee of Anhui University of Technology (Approval No.: YSXYLL2025001)**.**

Regarding the publicly available data utilized, we confirm that all sources were obtained through legitimate channels and that their use does not involve any infringement. Furthermore, all materials comply with ethical review standards, ensuring that the research does not violate personal privacy or copyright.

With respect to news images published by official media outlets used in this research, the individuals depicted are not research participants but rather participants in local ICH tourism experiences. These images are employed solely to illustrate the popularity of relevant ICH tourism projects and fully comply with ethical review requirements. In addition, all recognizable faces in the images have been blurred to ensure that no privacy concerns arise.

## 4. Analysis process and results

### 4.1 Open coding

Open coding is the process of decomposing, reviewing, comparing, conceptualizing, and categorizing data [86]. After multiple comparisons and analyses, this study identified 87 initial concepts. Subsequently, the attributes and intrinsic meanings of each initial concept were determined. Based on the semantic associations and logical relationships between the concepts, related concepts were grouped and integrated into the same category. Informed by relevant concepts from previous research on tourism experiences and experiential design theories, we accurately named the resulting categories, thereby identifying 15 preliminary categories: Professional Service Touchpoints, Experience Content Innovation, Scene Atmosphere Creation, Application of Modern Technology, Participation in Interactive Socialization, Sense of Participation, Sense of Wonder, Sense of Immersion, Sense of Resonance, Sense of Value, Cultural Awareness, Cultural Identity, Cultural Memory, Intangible Cultural Heritage Transmission, and Intangible Cultural Heritage Promotion. Partial open coding results are presented in Table 2. For instance, original textual descriptions such as “handcrafting,” “participating in these activities,” and “having the opportunity to experience it on stage” were summarized into conceptualized terms such as “firsthand experience” and “participation.” Drawing on Minnaert’s conceptual definition of tourism participation [87] and the description of “sense of participation” in experience economy theory, this section was preliminarily defined as the category “Sense of Participation.” For textual descriptions such as “feeling its unique charm,” “being deeply captivated by the silverware,” and “truly a delightful surprise,” these were summarized into conceptualized terms such as “uniqueness” and “magic.” Referring to the definitions of tourism wonder proposed by Tung [88]and Otto et al., this section was preliminarily defined as the category “Sense of Wonder.” For raw descriptions such as “the guide’s explanations were professional,” “the instructor’s guidance was professional,” and “tour services were attentive,” these were summarized into conceptualized terms such as “professional explanations” and “professional guidance.” Drawing on Stare et al.’s analysis of service touchpoints enhancing tourism experiences and Verhoef et al.’s classification of service touchpoints as experience drivers [89], this section was preliminarily defined as the category “Professional Service Touchpoints.” Establishing anchoring relationships be-tween themes and existing theoretical research on tourism experiences or profound experiences strengthens the theoretical foundation of the coding process, thereby enhancing the credibility of the coding results.

**Table 2:** Example of open coding.

Preliminary Category	Conceptualization	Example of Original Statement
Sense of Participation	firsthand experience	The silver bracelet I made with my own hands, though simple, carries meaning in every hammer strike.
	participation	Be able to appreciate and participate in these traditional folk activities up close, and experience the unique charm of folk culture.
	interact	In some Qinqiang performances, you can not only watch the performance, but also interact with the actors and even have the opportunity to experience it on stage.
	social contact	Jade carving enthusiasts, artists and collectors from all over the world gathered to exchange ideas.
	share	Share the clay figures you make on social media platforms.
Sense of Wonder	unique	Wear costumes made of Huangping batik and feel its unique wearing experience and cultural charm.
	attract	I was deeply attracted by those exquisite silver jewelry and felt the unique charm of Hmong culture.
	miraculous	I never expected the Wudang Mountain temple fair to have such high-tech features. Using AR to view the ancient appearance of the buildings is truly miraculous.
	surprised	It was a real surprise to see so many beautiful clay works and to be able to make your own.
	marvel	The copyists were able to accurately copy the brushwork, composition, color and other details of the original ancient paintings and calligraphy, which made people can’t help but marvel at the artistic attainments of the ancients and the superb skills of the copyists.
	wonderful	Every day in the scenic area, there is a “porcelain sound water parlor” performance, so that people can feel the wonderful experience of porcelain music performance.
	exceeded expectations	The dyeing has a wonderful gradient, the blues and greens are naturally articulated, and I am very pleased with the work I have made, which exceeds expectations.
	shock	Wuyuan Nuo dance is really too shocking, it feels like traveling back to ancient times.
Sense of Immersion	immerse	Away from the hustle and bustle of the city, immersed in the embrace of nature and the world of tea, it feels like all your worries have dissipated.
	tranquil and calm	Experience the art of thangka with peace and relaxation of the mind, and find a sense of inner peace.
	traveling through time and space	The experience of the blue printed fabric dyeing technique feels like traveling through time and space, allowing me to sense the cultural depth carried by this traditional craft.
	captivating and immersing	The charm of folk art is truly endless, captivating and immersing people in its beauty.
	focus	Experiencing tie-dye allows you to quiet your mind and focus on one thing, and it feels meaningful to watch the fabric gradually turn into a unique work in your hands.
	immerse themselves	Through online platforms or the virtual experience zones at the scenic spots, one can immerse themselves in the internal structure, spatial layout, and detailed craftsmanship of the stilted buildings.
	the soul had been purified	During the tea tasting process, I calmed my mind to experience the aroma, taste, and texture of the tea, feeling the tranquility and pleasure it brought, as if my soul had been purified.
	exhilarating	Wearing the headset and sitting in the dedicated seat, I enjoyed an exhilarating digital adventure journey.
Sense of Resonance	resonate with	The reverence and gratitude that fishermen feel towards the sea easily resonate with our inner emotions.
	proud	At the Yangliuqing Folklore and Culture Center, I experienced the making of a New Year’s painting for myself, and although my movements were clumsy, I felt very proud to finally see a New Year’s painting that I had made myself.
	inspired	During the experience of the Jun porcelain firing technique, I had in-depth communication and interaction with the local artisans. Their passion and dedication to Jun porcelain deeply inspired me.
	empathy	Demonstrating the characteristics of traditional muqam art while introducing modern cultural elements, it triggers a high degree of empathy.
	empathize deeply	The actors’ eye expressions, gestures, and other details were meticulously handled, allowing them to portray the emotions and inner world of the characters vividly, making us empathize deeply.
	moved	Watching the Nuo dance, I could feel the dancers’ love and dedication to traditional culture, and this emotion deeply moved me.
	admirable	With hands and feet, only 5–6 centimeters can be woven in a day, which is really an inch of brocade and an inch of gold! This traditional handloom weaving technique has condensed the wisdom and hard work of countless craftsmen, which is admirable.
Sense of Value	rewarding	This hands-on approach gave me a more intuitive feel for traditional craftsmanship, which was very rewarding.
	satisfied	I had a more intuitive understanding of the art of Anhui ink production, and this hands-on experience made me feel very happy and satisfied.
	accomplishment	At the Shan Shui Suzhou Embroidery Museum, we started with learning about the history of Suzhou embroidery, moved on to learning the basic stitches, and finally finished our own work, feeling very accomplished.
	belonging	This intimate human interaction gives us a sense of warmth and belonging.
	instructive	Not only did they learn, but they also developed an interest in traditional culture, which is a very good way to teach and have fun.
	responsibility	Realizing that I have a responsibility to preserve and pass on this valuable cultural heritage, this sense of mission lingers long after the experience is over.

### 4.2 Main axis coding

This study identified 15 preliminary categories through open coding; however, these categories are relatively independent, and the relationships between them are not yet clear [90], necessitating the use of main axis coding. Main axis coding involves further categorizing similar types of categories derived from open coding, organizing and establishing logical relationships, and connecting the different categories to form main categories [91]. This study extracted four main categories from the 15 preliminary categories: basic conditions, perception and evaluation, personal significance, and social significance (see Table 3).

**Table 3:** Main categories and corresponding concepts.

Main Categories	Corresponding Category	Include Concepts
Basic Conditions	Professional Service Touchpoints	professional presentation, professional guidance, well-organized, thoughtful services
	Experience Content Innovation	academic activities, parent-child interaction programs, co-creation activities, night tour programs, online activities, creative peripherals, innovative interpretation, personalized customization
	Scene Atmosphere Creation	scene atmosphere creation, virtual scene construction, cultural atmosphere shaping, activity atmosphere building, festival atmosphere baking, all-around feeling immersion, situational interaction
	Application of Modern Technology	holographic projection, virtual reality, 3D printing, naked eye 3D, interactive devices, 3D technology, light and shadow technology, internet technology, interactive technology
	Participation in Interactive Socialization	interactive activities, in-depth conversations, interactive exchanges, exchange and share, academic seminars
Perceptual Evaluation	Sense of Participation	firsthand experience, participation, interact, social contact, share
	Sense of Wonder	unique, attract, miraculous, surprised, marvel, wonderful, exceeded expectations, shock
	Sense of Immersion	immerse, tranquil and calm, traveling through time and space, captivating and immersing, focus, immerse themselves, the soul had been purified, exhilarating
	Sense of Resonance	resonate with, proud, inspired, empathy, empathize deeply, moved, admirable
	Sense of Value	rewarding, satisfied, accomplishment, belonging, instructive, responsibility
Personal Significance	Cultural Awareness	recognize, get to know, feelings, comprehend
	Cultural Identity	cherish, realize, compelled to admire, willingness to purchase, participate again, contribute yourself
	Cultural Memory	profound impression, unforgettable, precious memories, good memories, reminiscence
The Social Significance	ICH Transmission	collection and protection, systematic training, promotion of heritage and development
	ICH Promotion	promoting international impact, promoting traditional culture

### 4.3 Selective coding

**Fig 5:**
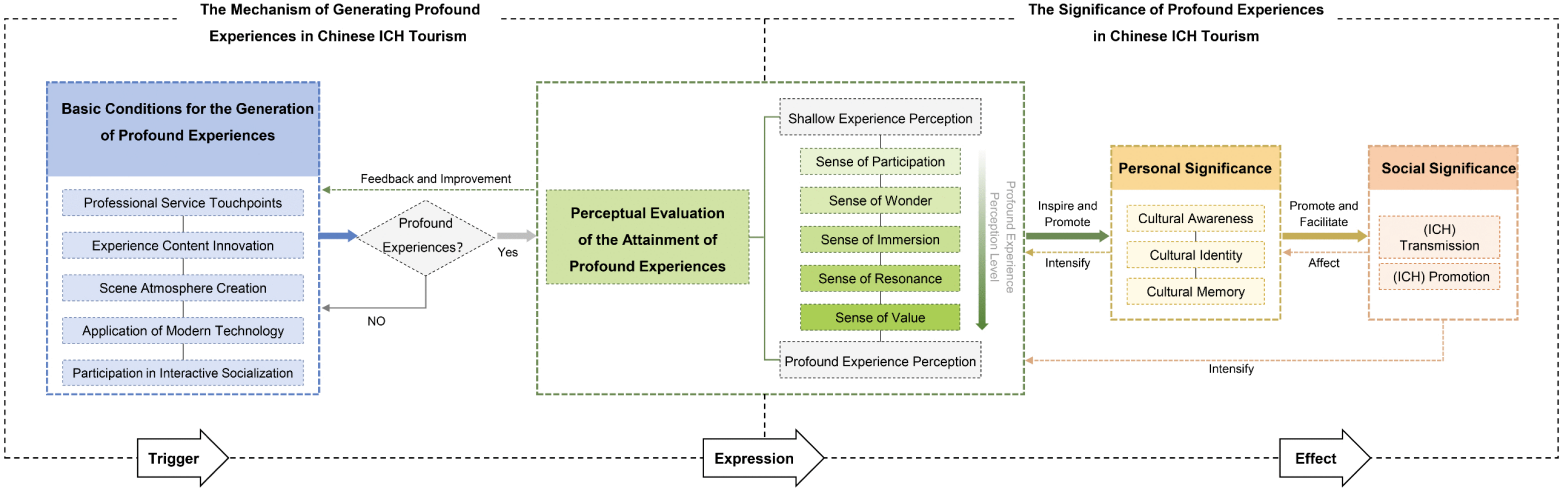
Theoretical model of profound experiences in Chinese ICH tourism. Based on the “trigger-expression-effect” storyline logic, the mechanism of generating profound experiences and the construction of meaning are explored.

Selective coding refers to the process of distilling core categories, further clarifying the logical relationships between main categories, core categories, and their corresponding concepts, and ultimately constructing a new theoretical model in the form of a narrative [92,93]. This study identifies the core category as “profound experiences in Chinese Intangible Cultural Heritage (ICH) tourism.” The connections between the main categories are explained based on the “trigger–expression–effect” narrative logic, constructing a theoretical model for the generation mechanism of profound experiences in Chinese ICH tourism (see Fig 5). Professional service touchpoints, experience content innovation, scene atmosphere creation, application of modern technology, participation in interactive socialization serve as foundational conditions for fostering the generation of profound experiences, triggering tourists to perceive and evaluate various profound experience outcomes such as a sense of participation, sense of wonder, sense of immersion, sense of resonance, and sense of value. The acquisition of profound experiences can enhance cultural cognition, cultural identity, and cultural memory in terms of personal meaning, while also contributing to the inheritance and dissemination of ICH in terms of social meaning. From the perspective of the influence of social meaning and personal meaning, social meaning further influences the sublimation of personal meaning. The acquisition of both social and personal meaning strengthens the intensity and sense of fulfillment associated with profound experience perception. The evaluation results of profound experience perception provide feedback on the fundamental conditions for their generation, thereby promoting their continuous improvement.

The “trigger–expression–effect” model of Profound Experiences constructed in this study first aligns with Kim et al.’s classical framework on memorable tourism experiences, which proposed a chain model of “antecedents–experience–outcomes.” This re-search validates the mediating role of experiential perception in tourism decision-making. Second, this study refines the perceptual outcomes of experiences. Building upon Kim et al.’s Memorable Travel Experience (MTE) scale, Gao et al.’s typology of experiences, Buonincontri et al.’s co-creation outcomes of tourism experiences, and Balaskas et al.’s cultural heritage experience evaluation scale, it demonstrates that profound experiences in ICH tourism are indeed composed of experiential outcomes such as a sense of participation, a sense of novelty, and a sense of value. Finally, this study expands upon previous research on meaning construction, advancing from memorable experiences to meaningful experiences. It emphasizes the value of experiences and explains how profound experiences generate cultural significance and social effects. Thus, the theoretical model summarized in this study remains faithful to China’s indigenous ICH tourism data. It not only clarifies the operational definitions of each element but also reveals the transformative pathway from foundational conditions to psychological perception within China’s ICH context. This fills a gap in existing ICH tourism research by providing a systematic experiential generation mechanism model for profound experiences.

### 4.4 Theoretical saturation test

Following the standard procedure of Grounded Theory, after the preliminary construction of the theoretical model, the researchers used the reserved text data related to the profound experiences of ICH tourism (accounting for approximately 1/6 of the total text) to verify the theoretical saturation of the model. The test results indicated that no new categories or inter-category relationships emerged in the newly added text data, and the core concepts were repeatedly observed. Therefore, it was concluded that the model passed the theoretical saturation test.

## 5. Model explanation

This study uses travelogues and reviews of profound experiences in China’s ICH tourism as its data source. Through open coding, main axis coding, and selective coding, it draws upon theoretical frameworks including experience economy theory, transformative tourism theory, cultural memory theory, factors of memorable travel experiences, and drivers of enhanced experience quality. Ultimately, it proposes a “theoretical model for profound experiences in China’s ICH tourism.” This model extends the application of tourism experience theories to the context of Chinese ICH tourism and systematically analyzes the generative mechanisms of profound experiences within this domain.

First, the model construction for the “trigger” stage draws upon experience economy theory and tourism experience theory. Integrating the textual data obtained in this study, it focuses on the drivers of experiential quality enhancement—including professional service touchpoints, experience content innovation, scene atmosphere creation, application of modern technology, and participation in interactive socialization—thereby proposing five basic conditions that facilitate the generation of profound experiences.

Second, in constructing the model for the “expression” stage, this study synthesizes relevant research and empirical data to categorize the perceptual evaluation of ICH tourism experiences into five progressive dimensions: sense of participation, sense of wonder, sense of immersion, sense of resonance, and sense of value. These perceptual evaluation outcomes draw upon descriptions of experience evaluation indicators from theories such as the experience economy, transformative tourism, and visitor happiness generation mechanisms. By linking these evaluation indicators to the Chinese context examined in this study, the research ultimately derives perceptual evaluation results for profound experiences in China’s ICH tourism.

Finally, in constructing the “effect” stage model, this study references Ballina F.’s [94] classification of tourism experience utility into individual and social dimensions. Based on data analysis, it concludes that acquiring profound experiences in ICH tourism generates both personal and social significance. Personal significance is summarized as cultural cognition, cultural identity, and cultural memory, while social significance encompasses ICH transmission and promotion.

In summary, this model construction not only expands the application scenarios of existing theoretical frameworks—including experience economy theory, transformative tourism theory, and cultural memory theory—but also integrates the specific context of China’s ICH tourism. It reveals the generative mechanisms and meaning-construction logic underlying profound experiences in Chinese ICH tourism.

### 5.1 Test the mechanism of generating profound experiences in Chinese ICH tourism

Throughout the entire process of ICH tourism, various factors such as professional service touchpoints, experience content innovation, scene atmosphere creation, application of modern technology, participation in interactive socialization, collectively contribute to enhancing tourists’ sense of fulfillment from their travel experiences. These factors serve as the “conditions” that foster the generation of profound experiences in ICH tourism.

(1) Professional Service Touchpoints

In the context of ICH tourism, touchpoints such as strategic inquiries, itinerary bookings, tour guide explanations, and catering and accommodation services all influence the overall experience of tourists over time. For example, De Keyser’s [95] TCQ terminology system indicates that touchpoints are regarded as core elements in shaping experiences. Systematic and professional touchpoint design enhances the operability and consistency of experiences. Similarly, Halb and Seebacher [96] propose that optimizing user experience depends on the systematic and quantitative management of key touchpoints. By identifying and evaluating highimpact touchpoints, continuous improvement in experience quality can be achieved. In particular, ICH tourism in China, with its emphasis on “intangible” culture, often necessitates professional interpretation and guidance to enable tourists to fully appreciate the aesthetic imagery and cultural significance of ICH. This, in turn, enhances the perceived value and satisfaction of the destination. For instance, “The museum not only boasts an extensive collection of exhibits but also provides professional interpreters who, through their explanations, allowed me to understand the intricate process and long history of copper carving techniques, which sparked my deep interest in this traditional craft.” (Case Number T28) The interpreters conveyed the history, techniques, and cultural implications of ICH, combining vivid language and body gestures to present the stories and cultural meanings behind the ICH, allowing visitors to fully experience the charm of this heritage.

(2) Experience Content Innovation

Experience content innovation addresses the issues of content homogenization and superficial experiences in ICH tourism. It accurately meets the diverse needs of tourists, stimulates their enthusiasm for in-depth participation, and serves as a crucial strategy to enhance the competitiveness of cultural perception. For example, Pencarelli [97] empirically demonstrated that content innovation driven by information and communication technologies plays a pivotal role in enhancing traditional tourism experiences, emphasizing the importance of integrating technology with innovative content. Furthermore, experiential content innovation should align with the dynamic context of social, environmental, and contemporary developments. As explored by Moon et al. [98], who investigated innovative travel approaches in the post-pandemic era, the concept of “cloud tourism” was introduced. They highlighted that immersive and empathetic virtual experiences can strengthen user perception and cultural identity. Such experience practices centered on content innovation have already yielded relevant cases in ICH tourism. For instance, “In the Yunjin Museum, children not only acquired knowledge but also developed an interest in traditional culture by observing looms and experiencing the weaving process firsthand.” (Case Number T37) Increasingly, tourists are eager to engage in various ICH activities to understand the historical narratives and emotional expressions embedded in ICH. In these activities, tourists are no longer passive observers, but are deeply involved in the experience. Another example is “Touring the Qinhuai River at night offers a unique experience. On the boat, one can enjoy the lights on both sides of the river and view the various light displays at the Fuzimiao Temple.” (Case Number T51) Visitors are also guided by ICH artisans to create traditional lanterns, from selecting materials and designing shapes to drawing patterns, thereby gaining a deeper understanding of the cultural meanings behind the lantern-making process.

(3) Scene Atmosphere Creation

ICH is often closely associated with specific historical, cultural, and social contexts. The creation of scene atmospheres can offer tourists a more authentic, environmental, and at-mospheric sense of ICH, thereby enabling a deeper understanding of the culture. Additionally, it establishes an environment or cultural setting that aligns with the ICH, encouraging tourists to experience its charm in an “immersive” manner. Li [99] et al. noted that nighttime tourism atmospheres contribute to creating memorable experiences by stimulating visitors’ feelings of pleasure and excitement. Building upon the theory of atmospheric aesthetics, Sun [100] et al. further emphasized that multidimensional interactions among physical environments, situations, and contexts can evoke emotional resonance and immersive experiences among tourists, validating the pivotal role of scene atmosphere creation in shaping high-quality tourism experiences. This approach of fostering profound experiences through atmosphere creation is particularly prominent in ICH tourism practices. For example, “Participating in small-scale Dongba cultural activities with local Naxi people and listening to the ancient Dongba sutra chanting in a room decorated with Dongba paintings, this form of in-depth interaction allows us to truly integrate into the cultural atmosphere of the Naxi people and experience the charm of Dongba paintings in all aspects.” (Case Number T11) Visitors are able to immerse themselves more easily and emotionally resonate with their surroundings, which facilitates the generation of profound experiences.

(4) Application of Modern Technology

The application of modern technology has expanded the ways in which tourists can obtain experiences, enabling them to encounter “incomparable” or “unprecedented” sensations. For example, Artificial Intelligence (AI) and Augmented Reality/Virtual Reality (AR/VR) technologies break the limitations of time and space, allowing tourists to experience ICH more intuitively and vividly. The creation and enhancement of cultural resources through advanced technology contribute to the activation of cultural heritage and the enrichment of knowledge and diversity within ICH tourism programs [101]. For instance, “I brought my child here, and he particularly enjoyed the VR experience, as if he had traveled to an ancient temple site and practiced with Taoist priests, to the point that he didn’t want to leave.” (Case Number T31) The application of modern technology in ICH tourism not only enhances the interactivity of the ICH experience but also promotes well-being and a positive improvement in mood, allowing tourists to feel greater enjoyment, surprise, immersion, and satisfaction. Scholars have pointed out that the digital revolution is propelling the tourism industry into the “Tourism 4.0” phase, which is centered on intelligence and sustainability [102], achieving an upgrade in tourism experiences that equally emphasizes digitalization and sustainability. Arifin [103] further demonstrates that modern technological applications optimize travel planning and experience processes through the integration of multiple technical means, significantly enhancing the quality of visitor experiences. Meanwhile, Anaya [104] reveals that technological applications influence three core dimensions of tourism experiences, and their impact on these experiences has shown significant fluctuations over time.

(5) Participation in Interactive Socialization

Participation in interactive socialization refers to the process where tourists communicate, share, and interact with ICH bearers, ICH interpreters, local residents, other tourists, and so on during ICH tourism. Through social interaction, tourists can, on the one hand, gain a deeper understanding of the historical origins, cultural connotations, and technical characteristics of ICH, thereby experiencing the culture more profoundly. For example, “Interacting with local Yi families and experiencing the traditional lifestyle of the Yi people, this in-depth interaction allowed me to understand Yi culture more comprehensively.” (Case Number T05) On the other hand, tourists can share their travel experiences and impressions with a wider audience through social media. For instance, “I made a small clay figure at the Huishan Clay Figure Experience Store, and after posting it to my friends, many of them liked it and inquired about it.” (Case Number T39) Briciu [105] indicated that social media platforms effectively promote tourists’ active participation and social interaction by facilitating user-generated and shared travel content, thereby deepening the participatory dimension of travel experiences. Concurrently, Luo [106] highlighted that information sharing and social interaction within online travel communities significantly stimulate users’ active engagement. This not only fosters communication and exchange among tourists but also enhances group belonging and cultural identity. Therefore, engaging in interactive socialization provides a platform for tourists to exchange views and share experiences, which facilitates a deeper connection to ICH culture. This interaction also allows tourists to develop a new understanding of ICH, deepening their three-dimensional knowledge and providing a more profound tourism experience.

To sum up, the service touchpoint serves as the starting point for the generation of profound experiences. The innovation of experience content stimulates tourists’ desire to explore and achieve a sense of unique experience. The creation of scene atmosphere adds emotional depth to tourists’ experiences through comprehensive immersion in the environment. The application of modern technology enhances cognitive breakthroughs and personalized experiences. Participation in interactive socialization further elevates the emotional and social value of the experience. These five conditions follow the path of “trust construction → interest stimulation → atmosphere reinforcement → technology value enhancement → value accumulation,” thereby deepening the acquisition of profound experiences, which together form a comprehensive system for the generation of profound experiences.

### 5.2 Perceptual evaluation of profound experiences in China’s ICH tourism

(1) Sense of Participation

The sense of participation refers to the feeling of immersion and engagement that tourists experience during ICH tourism, achieved through active involvement in ICH activities and the acquisition of ICH-related skills, both physically and mentally. “Participation” transforms tourists from traditional ‘spectators’ into “active participants” in ICH. It is only through direct involvement that one can gain a profound understanding of the content, essence, and significance of ICH. For instance, “This experience was amazing! Master Long Taiyang not only provided us with a theoretical lesson but also guided us step-by-step in making silver jewelry. The silver bracelet I crafted by hand, though simple, holds profound meaning in every hammer strike” (Case Number T03). Scholars have validated the crucial role of the “sense of participation” in cultural tourism experiences from multiple perspectives. De Bruin [107] introduced the concept of “participatory experience tourism”, emphasizing that tourists’ active engagement in activities drives the transition from passive consumption to active co-creation, thereby demonstrating the significance of the “sense of participation” in enhancing the value and meaning of tourism experiences. Haim-Litevsky [108] found that active participation enhances individuals’ sense of belonging and connectedness, thereby promoting well-being. Visitor participation in ICH tourism facilitates a shift from seeking material authenticity to pursuing personal experiential fulfillment. This enhances the authentic perception of the value of ICH [109], allowing visitors to cultivate a deeper sense of travel experience through their “cognitive” engagement with ICH.

(2) Sense of Wonder

The sense of wonder arises when tourists are profoundly captivated by remarkable ICH performances, folk activities, and skill demonstrations, experiencing surprise, novelty, and sensations that exceed expectations. Witnessing scenes rarely encountered in daily life, engaging in novel activities, and admiring exquisite folk crafts all deliver a powerful sensory impact on travelers. In this context, the sense of wonder is primarily conveyed through terms such as “unique,” “captivating,” “magical,” “surprising,” “admiring,” “wonderful,” “beyond expectations,” and “staggering.” For instance, “This experience provided me with a more intuitive understanding of traditional Chinese folk art and allowed me to feel the unique cultural charm of the Jiangnan water towns” (Case Number T39). “The aesthetic characteristics of Miao silver jewelry are so unique that I was deeply captivated by those exquisite pieces, experiencing the distinct allure of Miao culture” (Case Number T03). The unique allure of ICH often captures tourists’ attention, offering a more comprehensive, integrated, multi-sensory experience model [110], creating fresh, unique, or unforgettable experiences. Currently, in experiential perception evaluation research, many scholars regard the sense of wonder as a key indicator for generating visitor happiness [111,112]. Schinkel [113]further revealed that the sense of wonder can transcend familiar cognitive boundaries, triggering profound reflection and emotional resonance, thereby enhancing individuals’ understanding and identification with cultural experiences.

(3) Sense of Immersion

Sense of immersion refers to the mental state in which tourists are fully absorbed by an activity, deriving a high degree of enjoyment from it [114]. It can manifest through technology, content, environment, and atmosphere, encouraging visitors to deeply engage in ICH tourism. In tourism research, immersion encompasses four dimensions: embodied perception, emotional expression, temporal-spatial dislocation, and high levels of participation [115], promoting visitors’ sense of physical and mental fulfillment and generating positive psychological experiences. In this study, sense of immersion is primarily described using terms such as “immersion,” “peaceful tranquility,” “time travel,” “enchantment,” “focus,” “being there,” “spiritual purification,” and “thrilling.” For instance, “During the experience, it felt like traveling through time and space, experiencing the cultural heritage carried by this traditional craft” (Case Number T22). “Feeling the elegance and beauty of Kunqu opera, it felt like being immersed in the poetic imagery of ancient times, where the music lingers on” (Case Number T36).Extensive research has further validated the pivotal role of immersion in cultural tourism experiences. For instance, Wu [115] constructed and validated a tourist immersion scale encompassing embodied perception, emotional expression, temporal–spatial displacement, and high engagement, concluding that immersion is the core factor in creating memorable and extraordinary travel experiences. Irimiás [116] and colleagues further found that the more deeply tourists immerse themselves in a context, the more likely they are to “disconnect from the real world” and experience intense emotional resonance, indicating that immersion enhances the emotional depth and psychological engagement of experiences. Blumenthal [117] further revealed that tourists’ immersive experiences typically progress through three stages: “engagement trigger—engagement discourse—immersive state.” Immersion originates from dynamic participatory experiences and serves as a key mechanism for forming profound experiential perceptions and meaning construction. Overall, immersion reflects the visitor’s complete engagement in the ICH context, enhancing their deep under-standing of the cultural heritage. It also reflects the continuity, depth, and richness of the experience, helping sustain lasting happiness and enriching the visitor’s profound sense of immersion in ICH tourism.

(4) Sense of Resonance

A sense of resonance refers to a strong emotional connection and sense of identification that tourists experience when they are moved by the exquisite craftsmanship, beautiful handicrafts, and unique atmosphere embodied in ICH. Fundamentally, a sense of resonance goes beyond general artistic appreciation, craftsmanship admiration, or participation in activities. It is a cognitive resonance of “culture is me, I am culture.” Resonance also establishes a profound and lasting emotional bond, fostering cultural empathy between tourists and ICH. In this study, resonance is primarily expressed using terms such as “resonance,” “pride,” “inspiration,” “empathy,” “empathizing,” “moving,” and “admiration.” For instance, “The performance of Yue opera was so delicate; the actors’ expressions and gestures were filled with emotion, and I found myself crying along with them” (Case Number T20). “From them, I learned not only craftsmanship but also a spirit of perseverance and the transmission of traditional culture” (Case Number T43). Resonance reflects a deeper level of reflection by tourists on their own experiences in ICH tourism, achieved through the creation of “space,” “human interaction,” and “memory formation” [118]. This process also promotes deep cultural perception, emotional connection, and profound emotional resonance, significantly influencing the acquisition of tourists’ profound experiences. As Mohanty [119] and others have pointed out, emotional resonance enables individuals to establish profound emotional connections during experiences and translate them into actual participation intentions. Similarly, Allinson [120] observed in performance case studies that “resonance” can foster transformative connections between subjects and others. This phenomenon likewise exists in ICH tourism, where visitors undergo a psychological shift from “bystanders” to “identifiers” through emotional resonance during observation, participation, or interaction.

(5) Sense of Value

A sense of value refers to the psychological recognition that visitors gain through active participation and immersive experiences, leading to a sense of accomplishment and meaning. It also reflects cultural recognition of the social contributions and value of ICH tourism pro-jects. This sense of value can manifest through self-improvement, deep immersion in ICH, and the accumulation of happy memories from the journey. For example, “Experiencing Suzhou embroidery firsthand was truly fascinating. From learning basic stitching techniques to completing my own piece, I felt a great sense of accomplishment” (Case Number T18). A sense of value can also manifest as a deep understanding of the significant social contributions of ICH and an enhanced sense of cultural belonging among tourists. For instance, “Seeing this Tujia ethnic group’s woven fabric made me realize that this cultural tradition is worth pre-serving and that this cultural industry is worthy of further development” (Case Number T30). In this paper, the sense of value in profound ICH tourism experiences is primarily expressed using terms such as “meaning,” “satisfaction,” “achievement,” “belonging,” “education,” and “responsibility.” Zhu [121], Chen [122], and several other scholars have examined the formation mechanisms and dimensional characteristics of value perception through studies on tea culture tourism, red tourism, and related fields. They further indicate that value perception in cultural tourism primarily arises from educational enlightenment, emotional attachment, and social identification—findings that closely correspond to the dimensions of “education,” “achievement,” “belonging,” and “responsibility” proposed in this research. Creating meaningful experiences is a key pathway to fostering a sense of value. This, in turn, promotes positive emotional, spiritual, and value-based experiences for tourists, exerting a positive influence on their lives at both the individual and societal levels.

In summary, the sense of participation, wonder, immersion, resonance, and value in the perceptual evaluation of profound experiences in ICH tourism reflect a gradual progression from behavioral participation to emotional resonance and, ultimately, to the sublimation of value.

### 5.3 The significance of profound experiences in Chinese ICH tourism

Profound experiences in ICH tourism enhance tourists’ cultural awareness, cultural identity, and cultural memory at an individual level, while also promoting the inheritance and dissemination of ICH at a societal level. This not only has a positive impact on tourists’ cultural education, personal development, and value formation, but also holds significant im-plications for the preservation and dissemination of tourism destinations and ICH itself.

#### 5.3.1 Personal significance of profound experiences in Chinese ICH tourism.

(1) Cultural Awareness

Firstly, tourists are able to gain an in-depth understanding of the cultural connotations, historical background, and distinctive techniques of ICH projects. For example, “It felt like attending a vivid cultural lesson, which deepened my understanding of the traditional art of Huishan clay figurines” (Case Number T39). Secondly, tourists can enhance their under-standing of cultural content based on their personal insights, particularly through hands-on experiences, where they profoundly appreciate the unique value of ICH. For instance, “Through this experience, I not only learned the techniques but also gained a deeper under-standing of the cultural background of bronze sculptures” (Case Number T28). Finally, pro-found experiences in ICH tourism can stimulate tourists’ enthusiasm for exploring ICH culture, motivating them to actively participate in ICH activities, learn ICH techniques, and purchase ICH products, thereby further deepening their understanding of the cultural spirit of ICH. Research by scholars such as Kang [123] and Li [124], from perspectives including ICH education and traditional villages, indicates that cultural awareness serves not only as a foundational variable for experiential learning of ICH but also significantly influences individuals’ positive attitudes toward the experiential process and their construction of meaning. Additionally, Fan’s [125] research on tourists’ cross-cultural competence suggests that cultural awareness is not merely a singular process of knowledge acquisition; it is reflected not only in cognitive understanding but also extends to emotional resonance and behavioral engagement.

(2) Cultural Identity

The cultural identity cultivated through profound experiences in ICH tourism arises when visitors, after deeply engaging with ICH culture, develop resonance and recognition of ICH on cognitive, emotional, and value levels. First, visitors are captivated by the compelling performances and demonstrations of ICH techniques, recognizing the cultural symbolic significance embedded in such heritage. For instance, “Understanding the historical origins and development of Huangshan Maofeng tea, and experiencing the transmission and evolution of tea culture over the centuries” (Case Number T40). Second, through active participation in ICH activities, visitors are moved by the stories and atmosphere inherent in the heritage, fostering emotional resonance and a sense of cultural belonging. For example, “I deeply understood philosophical concepts such as ‘unity of heaven and humanity’ and ‘the interdependence and mutual restraint of yin and yang,’ and felt the depth and breadth of Tai Chi culture” (Case Number T42). Finally, visitors gradually come to comprehend the unique artistic, historical, cultural, and social values of ICH, embracing the worldview and values it embodies. For example, “These paintings are not only works of art but also witnesses to history and carriers of culture. We should protect and inherit these cultural heritage treasures”(Case Number T46). Lin [126] points out that “cultural experience” represents both the intrinsic pursuit and vivid embodiment of cultural identity. Through the cyclical learning process of “participation–description–interpretation–response,” it guides individuals from emotional engagement toward cultural identification. Furthermore, He [127] emphasizes that project activities and the cultural-emotional dimension within cultural experiences significantly promote visitors’ cultural identity, serving as crucial pathways for its formation. Cultural identity serves as the core mechanism for the sustainable development of ICH tour-ism, promoting greater respect and protection of ICH among visitors, thereby enhancing ethnic cultural identity and pride in ICH tourism [128].

(3) Cultural Memory

Cultural memory refers to the unique and profound embodied memory that tourists form after deeply engaging with and experiencing ICH content, which is perceptible, inheritable, and transmissible. According to Jan Assmann’s theory of cultural memory [129], texts, symbols, images, activities, and demonstrations within ICH serve as rich carriers for the formation of cultural memory. For example, “The tea-picking opera allowed me to gain a deeper under-standing of the folk culture and lifestyle of the people in southern Jiangxi” (Case Number T45). Profound experiences are a key means of promoting the formation of cultural memory. When tourists personally participate in ICH performances, handicraft making, and other activities, they undergo strong emotional and meaningful experiences that facilitate the creation of unique memories from ICH tourism. Li [130] noted that the cultural memory formed through creative participation and emotional resonance during cultural experiences not only deepens tourists’ understanding of local culture but also significantly enhances their destination attachment and satisfaction. As one visitor remarked,“These hands-on activities allow people to gain a deeper understanding of the local culture and characteristics, leaving me with a very unique and unforgettable travel memory” (Case Number T43). These experiential encounters are not static; rather, they are continuously re-constructed, reinforced, and strengthened in visitors’ memories, ultimately forming a complete memory impression that influences their cultural cognition and values. Cultural memory not only triggers visitors’ associations with ICH and promotes the construction of sustainable identity but also expands the “circle of friends” for the dissemination of ICH, driving its continued influence in daily life.

#### 5.3.2 The social significance of profound experiences in ICH tourism.

(1) Intangible Cultural Heritage (ICH) Transmission

ICH transmission occurs through various cultural activities that integrate “intangible cultural heritage + tourism,” facilitating the continuation and promotion of ICH [131]. On one hand, ICH tourism helps visitors gain a deeper understanding of the essence and allure of ICH culture, enhancing their sense of identification and resonance with ICH, and strengthening their awareness of the importance of actively participating in the in-heritance and protection of ICH culture. As an illustration, Song [132] used Chengdu lacquer art as a case study, pointing out that ICH transmission is not static preservation but a “living process” rooted in practitioners’ activities and social interactions. Its continuity relies on public participation and innovative safeguarding measures. For example, “As a site for the inheritance of ICH, Yunjin holds significant historical, cultural, and artistic value and is worthy of our protection and inheritance” (Case Number T37). On the other hand, ICH tourism allows certain ICH techniques at risk of extinction to receive in-creased resource investment for their continuation and development. This reflects the historical, cultural, and social value of ICH, thereby promoting its continuation, transmission, and development on a broader societal level. As Chung [133] revealed, the sustainability of ICH transmission relies not only on traditional master-apprentice systems but also on adapting to contemporary developments. This involves achieving cultural regeneration through diverse means such as technological innovation and media dissemination.

(2) Intangible Cultural Heritage (ICH) Promotion

ICH promotion involves the sustainable dissemination of the artistic forms, cultural connotations, and value concepts of ICH through ICH tourism activities. This not only allows tourists to experience the unique charm of ICH through tourism but also actively showcases the appeal of China’s excellent traditional culture. As Yin [134] observed, ICH promotion is essentially a ritualized process of co-constructing meaning. Through participatory interactions, tourists achieve cultural identity and shared values, thereby advancing the living transmission of ICH. Firstly, the cultural symbols, knowledge, and connotations associated with ICH attract visitors from diverse regions and cultural backgrounds. Through tourism experiences, visitors can develop a multi-dimensional cultural understanding and resonance, thereby enhancing the dissemination pathways and penetration of ICH culture. Secondly, profound experiences can promote tourists’ deeper understanding and recognition of ICH culture. Compared to traditional cultural display formats, experiencing and interacting with ICH culture firsthand can better stimulate tourists’ curiosity and achieve stronger cultural dissemination effects. In this process, the integration of digital technologies has provided diverse platforms for ICH promotion. Liu [135] pointed out that through multimedia approaches such as virtual experiences, interactive platforms, and holographic technology, the limitations of traditional promotion have been overcome. This has enhanced the immersive and interactive nature of ICH presentations, thereby in-creasing public engagement and awareness of cultural preservation [136]. Finally, tourism provides a rich platform for the dissemination of ICH culture, fostering ex-changes between various cultures. The deep integration of ICH and tourism can further drive the development of surrounding industries, offering new pathways and momentum for the inheritance, dissemination, and development of ICH culture.

In summary, the construction of the profound experiential significance of China’s ICH tourism reveals an interdependent and mutually reinforcing relationship between personal and social significance. On one hand, tourists’ recognition and identification with ICH pro-mote the transformation of individual experiential outcomes into drivers of cultural dis-semination, encouraging tourists to become practitioners of ICH protection through participation in its transmission activities. On the other hand, the transmission and dissemination of ICH have sustainable impacts on tourists’ cultural revisits and destination recommendations, motivating them to connect their personal contributions with cultural development. This fosters the sustainable development of culture from a higher social value dimension and creates meaningful, sustainable experiences for tourists.

## 6. Conclusions and outlook

### 6.1 Conclusions

(1) This paper clarifies the conceptual implications of profound experiences in ICH tourism. Profound experiences in ICH tourism refer to the emotional, value-related, and meaningful experiences that tourists gain through active participation in ICH tourism activities, allowing them to deeply perceive the spiritual essence of ICH culture. In the experience economy era, profound experiences represent a higher pursuit in ICH tourism and serve as one of the core drivers for the quality enhancement of cultural tourism. Tourists must deeply engage with the content of ICH and actively participate in ICH tourism activities to achieve profound experiences through cultural participation and interaction, thereby elevating the quality of their cultural experiences.
(2) This paper constructs a theoretical model for profound experiences in China’s ICH tourism. Using Grounded Theory and based on the “trigger-expression-effect” storyline logic, four main categories (basic conditions, perception and evaluation, personal meaning, and social meaning) and 15 corresponding subcategories are defined. Professional service touchpoints, experience content innovation, scene atmosphere creation, application of modern technology, participation in interactive socialization are the foundational conditions for achieving profound experiences in ICH tourism, exerting a positive influence on the realization of such experiences. The five dimensions of perceptual evaluation—participation, wonder, immersion, resonance, and value—not only describe the profound experiential feelings of tourists during ICH tourism activities but also serve as key criteria for assessing whether profound experiential tourism has been achieved. Profound experiences in Chinese ICH tourism not only influence individuals’ cognition, emotional connection, and value perception of ICH but also significantly impact travelers’ decisions to revisit ICH tourism destinations or recommend such experiences to others. The integration of ICH and tourism promotes the transmission and exchange of ICH, while enhancing travelers’ awareness of cultural preservation and transmission.

### 6.2 Theoretical and practical implications

This study holds significant theoretical value and practical implications. Theoretically, research on profound experiences in ICH tourism offers new perspectives and approaches for ICH tourism studies. First, this study extends the theory of profound experiences to the realm of ICH tourism, filling a gap in both domestic and international research on profound experiences in this area. Existing tourism experience models are predominantly applied to conventional tourism sectors (e.g., resort tourism, ecotourism), while studies focusing on profound experience models specific to ICH tourism remain limited. This paper defines the concept of profound experiences in ICH tourism and constructs a theoretical model that accounts for the unique characteristics of ICH. It organically integrates cultural cognition and ICH transmission with visitor experience, offering a new perspective for research on ICH tourism experiences. Second, this study refines the perceptual dimensions of experience within ICH contexts, transcending generic tourism experience scales. Existing research on tourism experiences commonly employs frameworks such as the Experience Economy 4E Model, the Memorable Tourism Experience Scale (MTE), and the Cultural Heritage Experience Evaluation Scale. However, these general models often fail to capture the cultural depth and spiritual essence inherent to ICH. This study explicitly identifies five perceptual dimensions of profound experiences in ICH tourism: sense of participation, sense of wonder, sense of immersion, sense of resonance, and sense of value. These dimensions reveal the relational structure of profound experiences in ICH tourism. This refined perceptual framework transcends generic tourism experience scales, expands the research paradigm for tourism experiences, and establishes a novel evaluation dimension system for ICH tourism studies. Third, this paper systematically analyzes the generative mechanisms of profound experiences in Chinese ICH tourism. Based on the trigger–expression–effect narrative logic, it proposes a theoretical model for pro-found experiences in ICH tourism. Unlike traditional tourism experience models, this framework integrates visitors’ cultural cognition processes, emphasizing the formation of cultural memory within ICH tourism and its influence on visitors’ emotions, values, and meanings. This model further reveals that profound experiences in ICH tourism are not merely visitors’ perceptual responses to ICH, but a dynamic process closely linked to ICH transmission.

In terms of practical application, the findings of this study contribute to meeting travelers’ deeper experiential needs in China’s ICH tourism, and provide concrete guidance for product design, service delivery, and cultural dissemination strategies in this field. First, regarding visitor experience, achieving profound experiences can significantly enhance travelers’ emotional connection to ICH and their understanding of its cultural value. Therefore, ICH tourism development should prioritize the design of immersive experiences by creating interactive scenarios and participatory elements aligned with tourists’ cultural cognition, enabling them to genuinely perceive the spiritual essence of ICH through engagement. Second, for managers, tourism managers should shift their focus from aesthetic experiences to tourists’ profound experiential needs, examining how basic conditions and perceptual evaluation guide the future design of profound ICH tourism experiences. Drawing on the cultural service touchpoint model, multitiered service points should be designed to move beyond traditional “spectator-based” formats, ensuring visitors have opportunities for profound engagement in ICH learning at every stage and enhancing their perceptual quality of heritage experiences. Combined with experience content innovation, immersive workshops, experience centers, and research-study bases for ICH skills should be developed to guide visitors in appreciating and engaging deeply with traditional crafts. Through environmental design and scene atmosphere creation, traditional craft processes or ancient settings can be recreated to enhance visitors’ sense of wonder and sense of immersion during experiences. The application of modern technology—such as virtual reality and augmented reality—can further innovate ICH presentation methods, transforming displays from static to dynamic formats and in-creasing experiential appeal. Authentic interactions between visitors and local residents or heritage bearers should be fostered to strengthen emotional connections, allowing visitors to appreciate cultural charm through engagement. By improving these basic conditions, visitor participation can be enhanced, experiential immersion deepened, unexpected surprises created, focus and immersion intensified, and emotional bonds strengthened to amplify cultural resonance. This ultimately promotes the social value of ICH and elevates visitors’ sense of value. In addition, these five perceptual evaluation dimensions can be used to assess whether visitors achieve profound experiential engagement. Third, in terms of the significance of ICH tourism, achieving profound experiences can help visitors better achieve cultural understanding, cultural identity, and cultural memory, thereby further enhancing the effectiveness of ICH transmission and dissemination.

### 6.3 Research limitations and prospects

This study has several limitations. First, the research data were obtained from tourist reviews in online travelogues, which inherently present certain limitations. Social media users are predominantly young and highly educated, potentially restricting the representativeness of the sample. Moreover, the sample collection lacked demographic stratification analysis, and as tourist reviews were the sole data source without cross-validation through field observations or in-depth interviews, the universality of the conclusions may be constrained. Future research should integrate data from multiple sources (e.g., field interviews, questionnaires) to enhance representativeness and reliability. Second, this study focuses on case research of ICH tourism experiences within the Chinese cultural context, without considering cross-cultural variations in perception. Tourists from different cultural backgrounds may perceive and evaluate ICH tourism experiences differently. Future research could collect data from cross-cultural tourists on the same ICH tourism case to examine perceptual differences in profound experiences among tourists from diverse cultural backgrounds, thereby improving the model’s generalizability. Third, although Grounded Theory provides profound qualitative insights, the validity of the findings still requires further quantitative verification. Future research should in-corporate quantitative methods for more in-depth analysis to test the specific effects of different perceptual dimensions on the profound experiences of ICH tourism.

Based on the findings of this study, future research can further explore the following areas: First, quantitative validation of the five dimensions of profound experiential perception. Future studies should employ quantitative methods (such as surveys and experimental research) to conduct confirmatory analyses of these five dimensions, further revealing their influence pathways on profound experiences in ICH tourism. Second, validation of the “trigger–expression–effect” mechanism across cultural contexts. As a form of cultural tour-ism, ICH tourism may present distinct triggering conditions and perceptual outcomes across different cultural backgrounds. Future research could compare profound experiences in ICH tourism by selecting similar heritage projects in China and other countries—such as China’s Yunjin brocade and Bangladesh’s Tangal sari weaving—to explore variations in visitor experiences across cultural contexts. Third, longitudinal studies on the formation of cultural memory in ICH tourism. ICH tourism not only shapes visitors’ immediate experiences but also exerts long-term effects on their cultural memory formation. Therefore, future research should conduct longitudinal investigations into cultural memory within ICH tourism, examining visitors’ long-term cognitive changes after participation and their profound impact on cultural identity. Such studies would provide sustained empirical foundations for the sustainable development of ICH tourism.

## Supporting information

S1 File NVivo12 profound experiences in Chinese ICH tourism. (ZIP)

S2 File The result of open coding. (DOCX)
