# Supplementary material for: Constructing a profound experience model of Chinese Intangible Cultural Heritage Tourism based on grounded theory
Source: PLoS One. 2026 Jun 11;21(6):e0351084. doi: 10.1371/journal.pone.0351084 (PMC13258011; doi:10.1371/journal.pone.0351084)
Supplement: S2 File — (DOCX) [file pone.0351084.s002.docx]

**Table.** The Result of Open Coding

| **Preliminary Category** | **Conceptualization** | **Example of Original Statement** |
| --- | --- | --- |
| Professional Service Touchpoints | professional presentation | The staff will make professional preparations and explain the history and artistic characteristics of Dongba painting to the tourists. (Case Number T11) |
|  | professional guidance | Purple clay pottery-making workshops are typically conducted under professional guidance by experienced ceramic artists. (Case Number T49) |
|  | well-organized | Sichuan Opera performances are usually organized by professional theaters or cultural institutions,the event was well organized to ensure the best viewing experience for the audience. (Case Number T50) |
|  | thoughtful services | The local thoughtful services made our stay especially comfortable, allowing us to fully relax and enjoy the joyful atmosphere of the Torch Festival. (Case Number T05) |
| Experience Content Innovation | academic activities | Wuyuan has launched a tea culture study tour, which is a series of academic activities that allow tourists to learn and experience tea culture. (Case Number T04) |
|  | parent-child interaction programs | Many parents brought their children to experience the parent-child interaction program of making Huizhou ink, which not only imparted valuable knowledge to the children but also strengthened the parent-child relationship. (Case Number T41) |
|  | co-creation activities | Participate in co-creation activities for the restoration of three-dimensional sculptures and carvings, and collaborate with local sculptors and carvers to create simple works. (Case Number T10) |
|  | night tour programs | The nighttime cultural tour programs at Yongding Tulou offer visitors a fresh travel experience, enriching their evening activities. (Case Number T01) |
|  | online activities | Qinqiang online live activities, enable real-time audience interaction, enhancing the viewing experience. (Case Number T19) |
|  | creative peripherals | From rice wine popsicles to rice wine face masks and other creative peripherals, these offerings have attracted a large number of young tourists. (Case Number T09) |
|  | innovative interpretation | By incorporating modern musical elements and dance movements, the ancient drum dance has received an innovative interpretation to better cater to the tastes of contemporary people. (Case Number T02) |
|  | personalized customization | Visitors can make personalized opera masks, which is a process from initial clumsiness to gradually mastering skills and becoming fully immersed in the fun. (Case Number T13) |
| Scene Atmosphere Creation | scene atmosphere creation | Using lighting and sound effects technology, combined with Tujia traditional music and natural sounds such as chirping insects, birdsong, and flowing water, an atmosphere of simplicity, tranquility, or liveliness is created. (Case Number T32) |
|  | virtual scene construction | Visitors can immerse themselves in the virtual experience zone on the online platform to explore the internal structure, spatial layout, and intricate construction techniques of these stilted houses. (Case Number T32) |
|  | cultural atmosphere shaping | Immerses visitors in a rich Bai ethnic cultural atmosphere, it allows guests to fully immerse themselves and authentically appreciate the cultural charm of Bai tie-dyeing. (Case Number T24) |
|  | activity atmosphere building | The Kun Opera Museum builded a activity atmosphere, and I was able to deeply appreciate the charm of Kun Opera. (Case Number T33) |
|  | festival atmosphere baking | On the day of the celebration, Xu Village was bustling like it was Chinese New Year. The entire village echoed with laughter and cheer, filled with a vibrant festive atmosphere. (Case Number T06) |
|  | all-around feeling immersion | Alongside the drumming, the event features distinctive Miao music and dance. While drumming, participants can also enjoy spectacular dance performances, immersing themselves in the all-around feeling of Miao culture. (Case Number T02) |
|  | situational interaction | Embark on a live-action, role-playing, situational interaction tour experience alongside NPCs as you journey through history, significantly enhancing visitor engagement and immersion. (Case Number T01) |
| Application of Modern Technology | holographic projection | Utilizing holographic projection technology, the performance showcases the distinctive features of traditional Mugham art while incorporating modern cultural elements, evoking deep emotional resonance among the audience. (Case Number T25) |
|  | virtual reality | Through 3D modeling and VR experiences, visitors can get an up-close look at the internal structure and construction process of stilt houses, gaining a deeper understanding of Tujia culture. (Case Number T32) |
|  | 3D printing | By showcasing the copying process through high-definition imaging technology and utilizing 3D printing to create copying tools, these technological methods not only enhance the precision of the copying process but also make the experience more intuitive and vivid. (Case Number T21) |
|  | naked eye 3D | The nighttime cultural tourism offerings at Yongding Tulou, such as the holographic stage and naked eye 3D light shows, provide visitors with a fresh travel experience and enrich their evening activities. (Case Number T01) |
|  | interactive devices | Digital media art interactive devices are truly magical. I felt as though I had stepped into a dreamlike world, experiencing the perfect fusion of Suzhou embroidery and modern technology. (Case Number T18) |
|  | 3D technology | Through 3D light-carving digital technology, an immersive world of light and shadow is created, allowing me to experience the boundless charm of Wuzhou culture in the realm between reality and illusion. (Case Number T26) |
|  | light and shadow technology | Some Sichuan Opera performances have incorporated modern stage light and shadow technology, making the performances more vivid and captivating. (Case Number T50) |
|  | internet technology | Internet technology allows users to send virtual flowers and leave messages, paying tribute to Sima Qian and expressing their condolences. (Case Number T06) |
|  | interactive technology | You can also scan the QR code or use NFC contactless interactive technology to listen to classic opera excerpts associated with the “Five-Color Face Masks” without touching the display. (Case Number T13) |
| Participation in Interactive Socialization | interactive activities | In some Qinqiang performances, you can not only watch the performance, but also interact with the actors and even have the opportunity to experience it on stage.This immersive interactive experience is incredibly fun and exciting. (Case Number T19) |
|  | in-depth conversations | Face-to-face conversations with Peking Opera performers, listening to their artistic journeys and insights into the art form. (Case Number T13) |
|  | interactive exchanges | Through interactive exchanges with Dongba artists and local Naxi people, this experience was enriched with a great deal of warmth. (Case Number T11) |
|  | exchange and share | Everyone gathered together out of shared interest in the craft of Wuyuan green tea production, exchanging and sharing their experiences and insights. (Case Number T04) |
|  | academic seminars | The scenic area hosts various ceramic cultural events and academic seminars, providing an important platform for the exchange and development of ceramic culture. (Case Number T15) |
| Sense of Participation | firsthand experience | The silver bracelet I made with my own hands, though simple, carries meaning in every hammer strike. (Case Number T03) |
|  | participation | Be able to appreciate and participate in these traditional folk activities up close, and experience the unique charm of folk culture. (Case Number T08) |
|  | interact | In some Qinqiang performances, you can not only watch the performance, but also interact with the actors and even have the opportunity to experience it on stage. (Case Number T19) |
|  | social contact | Jade carving enthusiasts, artists and collectors from all over the world gathered to exchange ideas. (Case Number T38) |
|  | share | Share the clay figures you make on social media platforms. (Case Number T39) |
| Sense of Wonder | unique | Wear costumes made of Huangping batik and feel its unique wearing experience and cultural charm. (Case Number T07) |
|  | attract | I was deeply attracted by those exquisite silver jewelry and felt the unique charm of Hmong culture. (Case Number T03) |
|  | miraculous | I never expected the Wudang Mountain temple fair to have such high-tech features. Using AR to view the ancient appearance of the buildings is truly miraculous. (Case Number T31) |
|  | surprised | It was a real surprise to see so many beautiful clay works and to be able to make your own. (Case Number T39) |
|  | marvel | The copyists were able to accurately copy the brushwork, composition, color and other details of the original ancient paintings and calligraphy, which made people can't help but marvel at the artistic attainments of the ancients and the superb skills of the copyists. (Case Number T21) |
|  | wonderful | Every day in the scenic area, there is a “porcelain sound water parlor” performance, so that people can feel the wonderful experience of porcelain music performance. (Case Number T15) |
|  | exceeded expectations | The dyeing has a wonderful gradient, the blues and greens are naturally articulated, and I am very pleased with the work I have made, which exceeds expectations. (Case Number T24) |
|  | shock | Wuyuan Nuo Dance is really too shocking, it feels like traveling back to ancient times. (Case Number T44) |
| Sense of Immersion | immerse | Away from the hustle and bustle of the city, immersed in the embrace of nature and the world of tea, it feels like all your worries have dissipated. (Case Number T40) |
|  | tranquil and calm | Experience the art of thangka with peace and relaxation of the mind, and find a sense of inner peace. (Case Number T35) |
|  | traveling through time and space | The experience of the blue printed fabric dyeing technique feels like traveling through time and space, allowing me to sense the cultural depth carried by this traditional craft. (Case Number T22) |
|  | captivating and immersing | The charm of folk art is truly endless, captivating and immersing people in its beauty. (Case NumberT45) |
|  | focus | Experiencing tie-dye allows you to quiet your mind and focus on one thing, and it feels meaningful to watch the fabric gradually turn into a unique work in your hands. (Case NumberT24) |
|  | immerse themselves | Through online platforms or the virtual experience zones at the scenic spots, one can immerse themselves in the internal structure, spatial layout, and detailed craftsmanship of the stilted buildings. (Case NumberT32) |
|  | the soul had been purified | During the tea tasting process, I calmed my mind to experience the aroma, taste, and texture of the tea, feeling the tranquility and pleasure it brought, as if my soul had been purified. (Case NumberT40) |
|  | exhilarating | Wearing the headset and sitting in the dedicated seat, I enjoyed an exhilarating digital adventure journey. (Case NumberT16) |
| Sense of Resonance | resonate with | The reverence and gratitude that fishermen feel towards the sea easily resonate with our inner emotions. (Case NumberT08) |
|  | proud | At the Yangliuqing Folklore and Culture Center, I experienced the making of a New Year's painting for myself, and although my movements were clumsy, I felt very proud to finally see a New Year's painting that I had made myself. (Case NumberT38) |
|  | inspired | During the experience of the Jun porcelain firing technique, I had in-depth communication and interaction with the local artisans. Their passion and dedication to Jun porcelain deeply inspired me. (Case NumberT43) |
|  | empathy | Demonstrating the characteristics of traditional muqam art while introducing modern cultural elements, it triggers a high degree of empathy. (Case NumberT25) |
|  | empathize deeply | The actors' eye expressions, gestures, and other details were meticulously handled, allowing them to portray the emotions and inner world of the characters vividly, making us empathize deeply. (Case Number T20) |
|  | moved | Watching the Nuo dance, I could feel the dancers' love and dedication to traditional culture, and this emotion deeply moved me. (Case Number T44) |
|  | admirable | With hands and feet, only 5-6 centimeters can be woven in a day, which is really an inch of brocade and an inch of gold! This traditional handloom weaving technique has condensed the wisdom and hard work of countless craftsmen, which is admirable. (Case Number T37) |
| Sense of Value | rewarding | This hands-on approach gave me a more intuitive feel for traditional craftsmanship, which was very rewarding. (Case Number T29) |
|  | satisfied | I had a more intuitive understanding of the art of Anhui ink production, and this hands-on experience made me feel very happy and satisfied. (Case Number T41) |
|  | accomplishment | At the Shan Shui Suzhou Embroidery Museum, we started with learning about the history of Suzhou embroidery, moved on to learning the basic stitches, and finally finished our own work, feeling very accomplished. (Case Number T18) |
|  | belonging | This intimate human interaction gives us a sense of warmth and belonging. (Case Number T33) |
|  | instructive | Not only did they learn, but they also developed an interest in traditional culture, which is a very good way to teach and have fun. (Case Number T37) |
|  | responsibility | Realizing that I have a responsibility to preserve and pass on this valuable cultural heritage, this sense of mission lingers long after the experience is over. (Case Number T02) |
| Cultural Awareness | recognize | This approach of seamlessly integrating art with daily life has allowed me to recognize the true value and meaning of art. (Case Number T46) |
|  | get to know | Moving from art back to reality, the exquisite artistic patterns woven into the brocade have allowed us to gain a deeper get to know the production, daily life, and cultural traditions of the Tujia people. (Case Number T30) |
|  | feelings | This interactive experience offers visitors a more intuitive understanding and feelings toward the art of jade carving, while also fostering respect for the craftsmanship spirit. (Case Number T38) |
|  | comprehend | Through this experience, I not only acquired the crafting techniques but also achieved a deeper comprehension of the cultural context behind bronze sculpture. (Case Number T28) |
| Cultural Identity | cherish | They can appreciate the cultural value and artistic charm embodied in purple clay pottery, thereby developing a deeper respect for and cherish towards this traditional craft. (Case Number T49) |
|  | realize | I deeply realized philosophical concepts such as ‘unity of heaven and humanity’ and ‘the interdependence and mutual restraint of yin and yang,’ and felt the depth and breadth of Tai Chi culture. (Case Number T42) |
|  | compelled to admire | As I admired the scene, I couldn't help but be compelled to admire this ancient yet vibrant culture. (Case Number T45) |
|  | willingness to purchase | At the intangible cultural heritage New Year painting market, I purchased two woodblock door god New Year paintings, intending to bring them back as gifts for my elders. (Case Number T34) |
|  | participate again | This Yue opera performance was incredibly creative, seamlessly blending tradition with modernity—it made me want to participate all over again. (Case Number T20) |
|  | contribute yourself | After experiencing it, people expressed their desire to contribute their own efforts to preserving and promoting Yangliuqing woodblock New Year paintings. (Case Number T46) |
| Cultural Memory | profound impression | Experiencing the perfect fusion of Tai Chi and natural landscapes truly offers a unique travel experience, leaving me with a profound impression of this place. (Case Number T42) |
|  | unforgettable | Through this experience, I gained a deeper understanding of the craftsmanship behind purple clay pottery. It was a truly unforgettable journey. (Case Number T27) |
|  | precious memories | Watching the sparks fly during the forging of Longquan swords was truly awe-inspiring, it would be one of the most precious memories of his journey. (Case Number T29) |
|  | good memories | Parents and children participated together in the making, sharing quality family time and creating good memories. (Case Number T41) |
|  | reminiscence | The Tea-picking opera provided me with a more comprehensive and profound understanding of this place, rendering my journey more meaningful and truly worthy of reminiscence. (Case Number T45) |
| ICH Transmission | collection and protection | We have come to recognize the uniqueness of Yunjin art and the importance of its preservation, which has sparked our desire to collect and protect it. (Case Number T37) |
|  | systematic training | Through systematic training, I not only mastered fundamental embroidery techniques but also gained a deep appreciation for the artistic value of Suzhou embroidery. (Case Number T18) |
|  | promotion of heritage and development | Wuyuan promotes the inheritance and development of tea culture by creating premium tea-themed study tours that allow visitors to learn and experience tea culture while sightseeing. (Case Number T04) |
| ICH Promotion | promoting international impact | The themed activities at Suzhou Embroidery Town were truly spectacular! Leveraging the CIIE as an international platform, they have promoted its international impact. (Case Number T18) |
|  | promoting traditional culture | They believe that they can better preserve and promote China's traditional tea culture, allowing this ancient craft to flourish with renewed vitality in the new era.(Case Number T40) |
